# A Reassessment of Copy Number Variations in Congenital Heart Defects: Picturing the Whole Genome

**DOI:** 10.3390/genes12071048

**Published:** 2021-07-08

**Authors:** Ilse Meerschaut, Sarah Vergult, Annelies Dheedene, Björn Menten, Katya De Groote, Hans De Wilde, Laura Muiño Mosquera, Joseph Panzer, Kristof Vandekerckhove, Paul J. Coucke, Daniël De Wolf, Bert Callewaert

**Affiliations:** 1 Center for Medical Genetics, Ghent University Hospital, Belgium and Department of Biomolecular Medicine, Ghent University, 9000 Ghent, Belgium; ilse.meerschaut@ugent.be (I.M.); sarah.vergult@ugent.be (S.V.); annelies.dheedene@ugent.be (A.D.); bjorn.menten@ugent.be (B.M.); laura.muinomosquera@uzgent.be (L.M.M.); paul.coucke@ugent.be (P.J.C.); 2 Department of Pediatric Cardiology, Ghent University Hospital, 9000 Ghent, Belgium; katya.degroote@uzgent.be (K.D.G.); hans.dewilde@uzgent.be (H.D.W.); joseph.panzer@uzgent.be (J.P.); kristof.vandekerckhove@uzgent.be (K.V.); daniel.dewolf@uzgent.be (D.D.W.); 3 Department of Pediatric Cardiology, Brussels University Hospital, 1090 Brussels, Belgium

**Keywords:** copy number variations, congenital heart defects, protein-coding genes, non-coding elements, topology associated domains

## Abstract

Copy number variations (CNVs) can modulate phenotypes by affecting protein-coding sequences directly or through interference of gene expression. Recent studies in cancer and limb defects pinpointed the relevance of non-coding gene regulatory elements such as long non-coding RNAs (lncRNAs) and topologically associated domain (TAD)-related gene-enhancer interactions. The contribution of such non-coding elements is largely unexplored in congenital heart defects (CHD). We performed a retrospective analysis of CNVs reported in a cohort of 270 CHD patients. We reviewed the diagnostic yield of pathogenic CNVs, and performed a comprehensive reassessment of 138 CNVs of unknown significance (CNV-US), evaluating protein-coding genes, lncRNA genes, and potential interferences with TAD-related gene-enhancer interactions. Fifty-two of the 138 CNV-US may relate to CHD, revealing three candidate CHD regions, 19 candidate CHD genes, 80 lncRNA genes of interest, and six potentially CHD-related TAD interferences. Our study thus indicates a potential relevance of non-coding gene regulatory elements in CNV-related CHD pathogenesis. Shortcomings in our current knowledge on genomic variation call for continuous reporting of CNV-US in international databases, careful patient counseling, and additional functional studies to confirm these preliminary findings.

## 1. Introduction

Copy number variations (CNVs) are DNA segments of one kilobase (kb) or larger, which are present at a variable copy number in comparison with the reference genome [1,2]. The introduction of chromosomal microarray analysis (CMA) in the late 1990s allowed the identification of small (below three megabases (Mb)) chromosomal imbalances down to resolutions of 100 kb. With the advent of massive parallel sequencing technologies, shallow whole genome sequencing (sWGS) entered the diagnostic field as a highly accurate, faster and cheaper alternative to CMA analysis.

CNVs can be directly causative of human diseases such as neurodevelopmental disorders and congenital malformations, or may contribute to the multifactorial risk for variable neurodevelopmental conditions including autism and intellectual disability, psychiatric disease, and congenital anomalies [2,3,4,5,6,7,8]. However, CNVs are also an important source of normal genetic variation [1,2], hence estimation of their pathogenicity is challenging.

Current clinical practice classifies CNV pathogenicity mainly based on size, parental inheritance, population frequency, known genotype–phenotype correlations, and protein-coding gene content of the CNV [9,10,11,12]. Despite these classification tools, many CNVs are classified as a CNV of unknown significance (CNV-US), with some of these CNV-US possibly being causative for the phenotype. In multifactorial disorders, in particular, common and often inherited variants might render a small effect to the phenotype, thus complicating the interpretation of CNV pathogenicity using the current criteria.

Moreover, recent advances in the identification of the molecular basis of limb defects and cancer revealed alternative CNV-associated disease mechanisms, affecting non-coding gene regulatory elements such as long non-coding RNAs (lncRNAs), enhancer elements, and disruption of the three-dimensional (3D) topologically associated domain (TAD) organization of the genome [13,14,15,16].

Congenital heart defects (CHD) are the most prevalent congenital malformation in live born children, and can occur isolated or be part of a syndromic constellation. Both isolated CHD (ICHD) and syndromic CHD (SCHD) can result from chromosomal aberrations including CNVs, single nucleotide variants, or multifactorial disease mechanisms [17,18,19,20,21,22,23,24]. So far, over 400 pathophysiological relevant CHD genes have been identified, including cardiogenic transcription factors such as NKX2.5 and members of the GATA or T-box families, cell signaling molecules involved in wnt, nodal, bmp, smoothened, notch, hippo, transforming growth factor β, fibroblast growth factor, ras, and vascular endothelial growth factor signaling, and chromatin-modifying genes [23,24,25]. Well-established CHD-related CNVs often affect such major cardiac developmental genes. Even though CNV analysis is relatively embedded in the diagnostic evaluation of CHD [8,26], studies reporting on the diagnostic value remain scarce and report yields between 12.8% and 18.5% [26].

Together, current knowledge on the molecular basis of CHD indicates an important contribution of transcriptional regulation in CHD. In line with this, a contribution of non-coding gene regulatory elements, as observed for limb defects and cancer, is conceivable. We sought preliminary evidence for such mechanisms in CHD performing a comprehensive retrospective analysis of reported CNV-US in a large in-house CHD cohort, focusing on potential interference with expression of pathophysiological relevant genes. More specifically, we evaluated recurrent CNV-US regions, the protein-coding gene content, lncRNA genes, and positional effects affecting TAD structures.

## 2. Materials and Methods

### 2.1. Selection and Characterization of the Cohort

Patients with a CHD born between January 2012 and July 2018 were recruited from the departments of pediatric cardiology and medical genetics of the Ghent University Hospital. CHD was defined as any defect in the structure of the heart or the great vessels present at birth. Acquired heart defects were excluded. For patients with atrial septal defects or patent ductus arteriosus, only those requiring a therapeutic intervention (surgical correction or percutaneous intervention) were retained.

Patients with available molecular karyotyping were included. Those patients with CHD and an underlying diagnosis (i.e., explanatory aneuploidy, a known monogenic condition, or prenatal conditions such as medication use, toxins or infection) were excluded.

The cohort was subdivided into SCHD and ICHD, based on the available clinical information. SCHD were defined as CHD that are associated with at least one additional major malformation and/or multiple minor physical anomalies and/or intellectual disability. Major malformations were considered malformations having a significant medical, social or cosmetic consequence, requiring medical intervention (e.g., neural tube defects, cleft palate, diaphragmatic hernia). In contrast, minor anomalies pose no significant health problem and have limited social or cosmetic consequences (e.g., preauricular tags, clinodactyly). Per the definition, we classified all patients with isomerism as SCHD cases.

This retrospective study was approved by the Ethics Committee of Ghent University Hospital (EC UZG 2016/0133).

### 2.2. Molecular Karyotyping

Molecular karyotyping was performed by CMA or sWGS. CMA was carried out using array comparative genome hybridization (arrayCGH) using a 180 k oligonucleotide array (Agilent Technologies, Santa Clara, CA, USA) with a genome-wide resolution of 100 kb. The reported breakpoints of the CNV regions identified by CMA represent the affected oligos, and thus the minimal affected CNV regions. sWGS was performed on Hiseq3000 sequencer (Illumina, San Diego, CA, USA) with an average genome-wide coverage of 0.1× to 1×. The minimal resolution of sWGS is 100 kb. For library preparation the NEXTflex Rapid DNA sequencing kit (Perkin Elmer, Waltham, MA, USA) was used.

CNV data evaluation and interpretation was performed using in-house developed software tools arrayCGHbase [27], Vivar [28] and WisecondorX [29]. CNVs that passed the quality control were further interpreted based on their size, overlap with known disease loci (the human morbid map), presence in control populations (Database of Genomic Variants), gene content, and additional relevant information from an extended literature search. Known pathogenic CNVs, CNV-US ≥ 100 kb, and CNV-US < 100 kb containing one or more protein-coding genes were reported (irrespective of the performed CNV analysis technique), and thus further evaluated in this retrospective study. All CNVs were mapped to the reference genome GRCh37(hg19).

### 2.3. Descriptive Statistics and Study of CNV-US

We performed a descriptive statistical analysis of the clinical characteristics of the patients and the results of their CNV analysis, in the total cohort, as well as in the SCHD and ICHD subgroups. Reported CNVs were listed and subdivided into pathogenic CNVs and CNV-US, based on the list of ClinGen Pathogenic CNV regions (https://dosage.clinicalgenome.org/pathogenic_region.shtml, accessed on 28 February 2021) [30]. CNVs for which a causal relationship with CHD is unclear were considered CNV-US.

CNV-US were evaluated for:

1. Overlap with ClinGen Pathogenic CNV regions (see above) or ClinGen Dosage Sensitive regions (https://search.clinicalgenome.org/kb/gene-dosage, accessed on 28 February 2021) [30].
2. Overlap with other CNV-US within the study cohort.
3. The protein-coding gene content based on NCBI RefSeq Select genes (Updated Annotation Release 105.20190906), extracted from the UCSC Genome Browser using the Table Browser [31].
4. Non-coding gene-regulatory elements contained within the CNV-US. LncRNA genes were extracted from the lncipedia high confidence set (hg19) (https://www.lncipedia.org/, accessed on 8 December 2020) [32]. Human VISTA enhancer elements were downloaded from the VISTA enhancer database (https://enhancer.lbl.gov/, accessed on 7 December 2020) [33].
5. Interference with the genomic TAD structure, based on the TAD boundaries file that was provided by J. Dixon to the developers of ClinTAD (https://www.clintad.com/, accessed on 4 January 2021) and publicly available on github [34,35]. This TAD boundaries file was generated using H1 human embryonic stem cells, chromosome build GRCh37, a bin size of 40 kb, and a window size of 2 Mb. To evaluate the potential disruption of TAD-related gene-enhancer interactions, we considered the protein-coding gene content (NCBI RefSeq Select genes) and the human VISTA enhancer elements within the relevant TAD domains.

### 2.4. Data Browsing

#### 2.4.1. Dosage Sensitivity of Protein-Coding Genes

Dosage sensitivity of affected protein-coding genes was evaluated using ClinGen Dosage Sensitivity Curations for haploinsufficiency and triplosensitivity (https://search.clinicalgenome.org/kb/gene-dosage, accessed on 31 March 2020) [30], and haploinsufficiency parameters: %HI scores (Decipher Downloads—Haploinsufficiency Predictions version 3) [36], pLI scores and upper bounds of observed/expected (o/e) confidence interval (gnomAD version 2.2.1 download—pLOF Metrics by Gene) [37]. Cutoffs used as a positive argument for haploinsufficiency were a %HI score < 10%, a pLI score > 0.90 and/or an upper bound of the o/e confidence interval < 0.35.

#### 2.4.2. Expression Data of Protein-Coding and lncRNA Genes

Expression data of genes during development of the human heart were obtained from a publicly available RNA-seq time-series dataset covering the development of seven organs, including the heart (http://www.ebi.ac.uk/, accession number E-MTAB-6814, accessed on 12 December 2020) [38]. All genes with an expression threshold of two transcripts per million (TPM) in heart tissue in at least one developmental stage between “four weeks post conception” and “neonate”, were considered as being expressed in developing heart tissue.

#### 2.4.3. Protein-Coding Gene Function Annotation

Protein-coding gene function was explored, evaluating the presence of the protein-coding genes in gene lists that might link them to heart development and/or CHD pathogenesis. These gene lists include an in-house CHD panel of 471 known or potential CHD genes (https://www.cmgg.be/assets/bestanden/GENPANEL-CHD.pdf, accessed on 1 April 2020), 1638 transcription factors [39], and 3430 genes linked to CHD-related Gene Ontology terms (wnt signaling pathway GO:0016055, nodal signaling pathway GO:0038092, bmp signaling pathway GO:0030509, smoothened signaling pathway GO:0007224, notch signaling pathway GO:0007219, hippo signaling GO:0035329, transforming growth factor β receptor signaling pathway GO:0007179, fibroblast growth factor receptor signaling pathway GO:0008543, ras protein signal transduction GO:0007265, vascular endothelial growth factor signaling pathway GO:0038084, sarcomere GO:0030017, cilium GO:0005929, histone modification GO:0016570, and chromatin remodeling GO:0006338) (Gene Ontology Release version 2020-03-24) [40]. These gene lists can be found in Supplementary Table S1.

## 3. Results

### 3.1. Study Cohort and Clinical Characteristics

The study cohort consisted of 290 CHD patients. Twenty patients (19 SCHD patients and one ICHD patient) were excluded because alternative genetic tests revealed a genetic defect explanatory for the CHD phenotype. These included trisomy 13, trisomy 21, monosomy X, mosaic trisomy 12, derivative 18, derivative 22, 8p deletion/duplication syndrome, marker chromosome 22 (cat eye syndrome), a 10 Mb 13q33.2 deletion, five patients with Noonan (-like) syndrome, three patients with CHARGE syndrome, one patient with Kabuki syndrome, one patient with Cornelia de Lange syndrome, and one patient with an *ELN* mutation.

The remaining 270 CHD patients were included in this study. Hence, the cohort including 87 SCHD patients (32.2%) and 183 ICHD patients (67.8%). The sex ratio was 158 boys (58.5%) to 112 girls (41.5%) (56 boys to 31 girls in SCHD; 102 boys to 81 girls in ICHD). The heart defects are listed in Supplementary Table S2. It is of note that all 37 patients with transposition of the great arteries were ICHD patients.

In the SCHD group, two patients harbored a chromosomal defect unrelated to the CHD: a triple X and a mosaic i(Y) (p10) and were included in the further analysis. In the ICHD group, two patients harbored a chromosomal defect unrelated to the CHD: XYY and .ish der(Y) and were included in the further analysis.

### 3.2. CNV Analyses

CNV analysis was performed by CMA in 227 (84.1%) patients and by sWGS in 43 (15.9%) patients

Seventeen patients of the SCHD group (19.5%) harbored a pathogenic CNV that was regarded causative for the CHD, including seven proximal 22q11.2 deletions (MIM 188400), one central 22q11.2 deletion, one distal 22q11.2 deletion (MIM 611867), one proximal 22q11.2 duplication (MIM 608363), four 7q11.23 deletions (MIM 194050), one 17q21.31 deletion (MIM 610443), one 17p11.2 duplication (MIM 610883), and one 4p16.3p15.32 deletion (MIM 194190) (see Supplementary Table S3). These pathogenic CNVs were all reported as pathogenic in the original lab reports, and hence not reclassified based on this study. Thirty-six patients with SCHD (41.4%) harbored a total of 47 CNV-US (min. 1, max. 4, median 1). One girl presented with a Xp22.31 deletion. Deletions of this region are associated with X-linked ichthyosis in males, but have not yet been associated with CHD. Therefore, we considered it as a CNV-US in the further analysis. The remaining 34 patients with SCHD (39.1%) had a normal CNV analysis.

None of the patients in the ICHD group had a CNV that was considered causative for the CHD. Two patients had a CNV for which the association with CHD remains under debate, a proximal 16p11.2 microdeletion and a 16p13.11 microdeletion, and were therefor considered as CNV-US in the further analysis. As such, 69 of the 183 patients with ICHD (37.7%) harbored a total of 91 CNV-US (min. 1, max. 3, median 1). The remaining 114 patients with ICHD (62.3%) had a normal CNV analysis.

**Figure 1:**
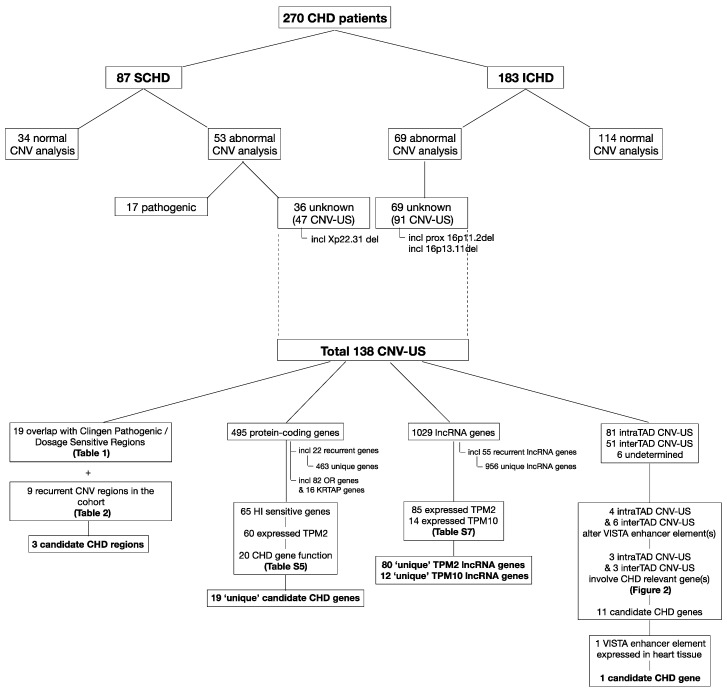
Structured overview of the study results. The upper part of the figure gives an overview of the included patients and the result of the copy number variation (CNV) analyses. The lower part of the figure depicts the flow and the final results of the explorative analyses performed in the CNV of unknown significance (CNV-US). CHD = congenital heart defects; SCHD = syndromic CHD; ICHD = isolated CHD; HI = haploinsufficiency; TPM = transcripts per million; TAD = topologically associated domain.

The included patients and the results of their CNV analyses are depicted in Figure 1.

### 3.3. Comprehensive Reassessment of CNV-US

#### 3.3.1. CNV Descriptives: CNV Type, Size and Parental Inheritance

The total study cohort contained 138 CNV-US for a comprehensive reevaluation, 47 CNV-US in the SCHD subgroup and 91 CNV-US in the ICHD subgroup. An overview of the CNV-US is given in Supplementary Table S4.

The CNV-US include 42 copy number losses (30.4%) (41 deletions and 1 mosaic deletion) and 96 copy number gains (69.6%) (90 duplications and six triplications). The proportion of copy number losses versus copy number gains is comparable between the SCHD and ICHD groups, respectively, 13 losses (27.7%) (including one mosaic deletion) and 34 gains (72.3%) (33 duplications and one triplication) in the SCHD group and 29 losses (31.9%) and 62 gains (68.1%) (57 duplications and five triplications) in the ICHD group.

The average length of the reported CNV-US is 417.76 kb (min. 6.66 kb, max. 5.08 Mb). The copy number gains are on average 394.99 kb (min. 6.66 kb, max. 2.06 Mb) and the copy number losses are on average 314.56 kb (min. 18.81 kb, max.5.08 Mb). In the SCHD subgroup the average length of CNV-US is 409.05 kb (min. 19.83 kb, max. 2.06 Mb). In the ICHD subgroup the average length of CNV-US is 422.25 kb (min. 6.66 kb—max. 5.08 Mb).

For the 105 patients harboring at least one CNV-US, parental DNA was available for segregation analysis for 66 patients (62.9%). As such, parental inheritance was shown for 81 of the 138 CNV-US (58.7%). Six CNV-US (4.3%) occurred de novo. For the remaining 51 CNV-US (37%) information on parental inheritance was unavailable. The de novo CNV-US occurred in two patients with SCHD and four patients with ICHD, and included three copy number losses (two germline deletions and one mosaic deletion) and three copy number gains (two duplications and one triplication). The segregation data are included in Supplementary Table S8.

It is of note that X-linked CNVs in females may be particularly challenging to interpret due to possibly skewed X-inactivation.

#### 3.3.2. Overlap with Known Dosage Sensitive Regions and Recurrence in the Study Cohort

In addition to the previously mentioned 16p11.2, 16p13.11, and Xp22.31 deletions, 16 other CNV-US show (partial) overlap with ClinGen Pathogenic and/or Dosage Sensitive CNV regions (Table 1). The contribution of 15q11.2 [41,42], 16p11.2 (MIM 611913), and 16p13.11 [43] microdeletions to CHD are debated. For the microdeletions in 2q13 [44] and 10q22.3q23.2 [45] and the duplication in 17p11.2 (MIM 610883), the suggested CHD candidate genes (respectively *BCL2L11*, *BMPR1A*, and *RAI1*) are not present in these CNV-US regions.

Nine CNV regions show recurrence within the CHD cohort, of which two (Xp22.31 and 15q11.2 recurrent region) overlap with one of the ClinGen Pathogenic or Dosage Sensitive regions (Table 2). Based on the smallest region of overlap (SRO) of the recurrent CNV-US regions, the occurrence of comparable CNVs in Decipher (https://decipher.sanger.ac.uk/, accessed on 22 March 2021) [36] and/or the Database of Genomic Variants (DGV) (http://dgv.tcag.ca/dgv/app/home, accessed on 22 March 2021) [46], we retained the following three SRO as potential interesting candidate regions for CHD: chr9:107409509-107729796, chr21:47591379-47671404, and chrX/Y:61091-437220.

#### 3.3.3. Protein-Coding Gene Content

Based on the protein-coding gene content, the 138 CNV-US can be subdivided into 116 coding CNV-US and 22 non-coding CNV-US. The 116 coding CNV-US affected a total of 495 NCBI RefSeq Select genes, ranging from 1 to 79 NCBI RefSeq Select genes per CNV-US (mean 4.27, standard deviation 9.21, median 2). Of these, 162 genes were entirely deleted by a CNV-US (seven genes resided in a mosaic deletion), 227 were duplicated, 9 were triplicated, and 97 genes were disrupted by a CNV breakpoint and thus partially deleted (25, including one mosaic), duplicated (67) or triplicated (5). Twenty-two genes were recurrently affected in two or more CNV-US, resulting in 463 uniquely affected genes. In the further results, we considered the recurrent genes as separate counts, because these were sometimes disrupted differently in different CNV-US. The 82 olfactory receptor (*OR*) genes and the 16 keratin associated protein (*KRTAP*) genes affected in the CNV-US, were disregarded for further evaluation in the context of CHD.

Evaluation of dosage sensitivity indicated that for 65 genes haploinsufficiency may be harmful. These included 24 deleted genes and 41 genes disrupted by a CNV breakpoint (10 partial gene deletions, 28 partial gene duplications and 3 partial gene triplications). Especially deletions and partial deletions are more likely to be disruptive than duplications or triplications. None of the duplicated genes are known to be triplosensitive. Of these haploinsufficiency sensitive genes, 60 genes (23 deleted genes and 37 breakpoint genes) show expression in developing human heart, with 20 genes (9 deleted genes (including one recurrent gene) and 11 breakpoint genes) (*ARFGEF2*, *AUTS2*, *CHAMP1*, *CHD8*, *CYFIP1* (2X), *FERMT2*, *ITCH*, *KMT2C*, *MAPK3*, *MAZ*, *MYH11*, *NASP*, *NPAS2*, *PIK3C3*, *PKNOX1*, *TIA1*, *TJP1*, *TRIM28*, *ZBTB21*) having a function that can be related to heart development or CHD pathogenesis. These genes are present in 18 different CNV-US, of which three occurred de novo (containing *AUTS2*, *CHD8* and *TRIM28*). These genes and their potential link to heart development and/or CHD (including the most relevant results from an additional Pubmed search looking for associations of these genes with (congenital) heart disease and/or heart development) are represented in Supplementary Table S5 [47,48,49,50,51,52,53,54,55,56,57,58,59,60,61,62,63].

An overview of the dosage sensitivity, expression in developing human heart, and gene function related to heart development or CHD of all 495 NCBI RefSeq Select genes in the CNV-US is given in Supplementary Table S6.

#### 3.3.4. lncRNAs

The 138 CNV-US contained a total of 1029 lncRNA genes, with a range from 0 to 193 lncRNA genes per CNV-US (mean 7.46, standard deviation 17.25, median 4). Of these, 425 resided in deletions (eight in the mosaic deletion), 576 in duplications, and 28 in triplications. Fifty-five lncRNA genes appear in more than one CNV-US, resulting in a total of 956 uniquely affected lncRNA genes. In the further results, the recurrent lncRNA genes were all counted separately.

Evaluation of expression in developing human heart tissue (after conversion to ENSG IDs) indicated that 85 of all lncRNA genes in the CNV-US (one lncRNA gene occurred three times and three lncRNA genes occurred twice, thus 80 unique lncRNA genes) reach at least 2 TPM in the developing human heart, and 14 lncRNA genes (one lncRNA gene occurred three times, thus 12 unique lncRNA genes) even reach 10 TPM. The 85 lncRNA genes with expression in developing human heart are present in 38 of the 138 CNV-US. An overview of these lncRNA genes is given in Supplementary Table S7.

#### 3.3.5. Enhancers and Interference with TAD-Related Gene-Enhancer Interactions

Of the 138 CNV-US, 81 are intraTAD CNVs (25 deletions, 52 duplications and four triplications) and 51 are interTAD CNVs (13 deletions, 37 duplications and one triplication). Six CNV-US occur in chromosome regions where no clear TAD regions/boundaries are determined (four at a telomere end of a chromosome and two at the Y chromosome).

**Figure 2:**
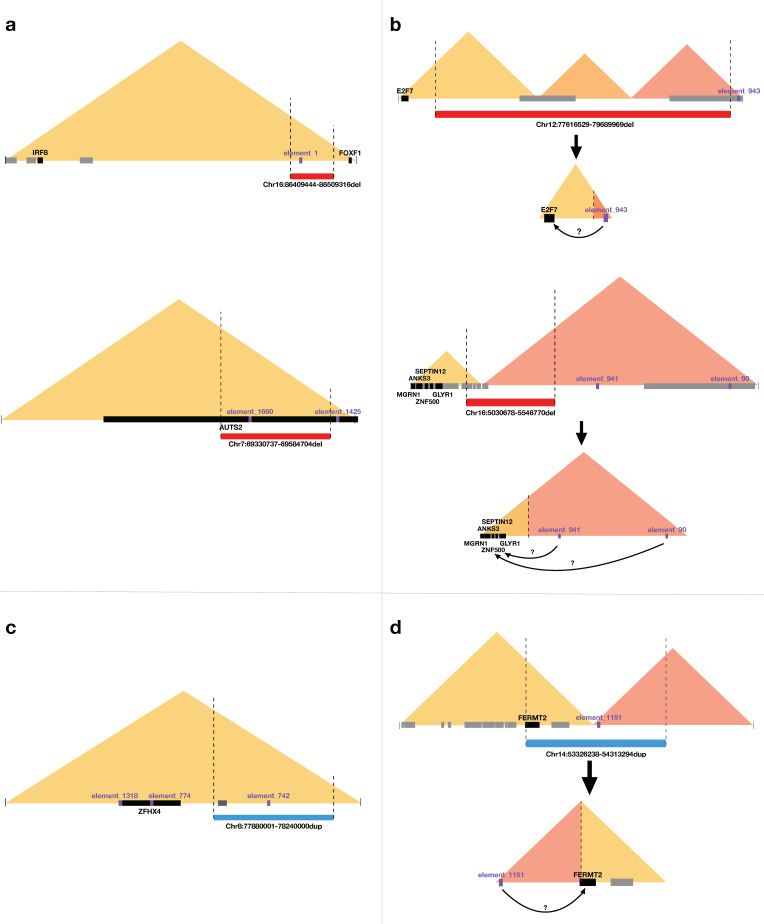
Schematic representation of the intraTAD deletions (**a**), interTAD deletions (**b**), intraTAD duplications (**c**), and interTAD duplications (**d**), potentially altering gene-enhancer interactions relevant for the congenital heart defects (CHD). The deletions are shown as red bars. The duplications are shown as blue bars. The triangles reflect the topologically associated domain (TAD) structure of the locus. CHD candidate protein-coding genes are depicted in black, other protein-coding genes within the TAD are depicted in grey, VISTA enhancer elements are depicted in purple. It is of note that only for VISTA element 1151 expression in the heart was confirmed (in transgenic mice at E11.5).

Two intraTAD deletions and two intraTAD duplications alter the dosage of a VISTA enhancer element (elements 1, 1660, 92, and 742), thereby potentially impacting the expression of target gene(s) within the TAD. Four of these genes are considered potentially CHD-relevant, occurring in two intraTAD deletions (*FOXF1*, *IRF8*, *AUTS2*) and in one intraTAD duplication (*ZFHX4*). A schematic representation of these latter three CNV-US and their potential impact on these gene-enhancer interactions is given in Figure 2. It should be noted, however, that none of the VISTA enhancer elements involved here are shown to be expressed in the heart (evaluated in transgenic mice at embryonic day 11.5 (E11.5)).

Four interTAD deletions and two interTAD duplications result in the formation of a neoTAD, involving a VISTA enhancer element (elements 943, 90, 941, 799, 305, 852, 1151, 1320, and 184), thereby potentially altering the expression of target gene(s) within the neoTAD. Seven of these genes are of interest in relation to CHD pathogenesis. They are related to two interTAD deletions (*E2F2*, *ANKS3*, *GLYR1*, *MGRN1*, *SEPTIN12*, *ZNF500*) and one interTAD duplication (*FERMT2*). However, the latter can only be if the duplication occurred in tandem. A schematic representation of the latter three neoTADs and the potential novel gene-enhancer interactions is given in Figure 2. It is of note that only one of the VISTA enhancer elements (element 1151) was shown to be expressed in the heart (evaluated in transgenic mice at E11.5).

An overview of the relevant NCBI RefSeq Select genes in the CNV-US related TADs and their gene function related to heart development or CHD is added to Supplementary Table S6.

**Figure 3:**
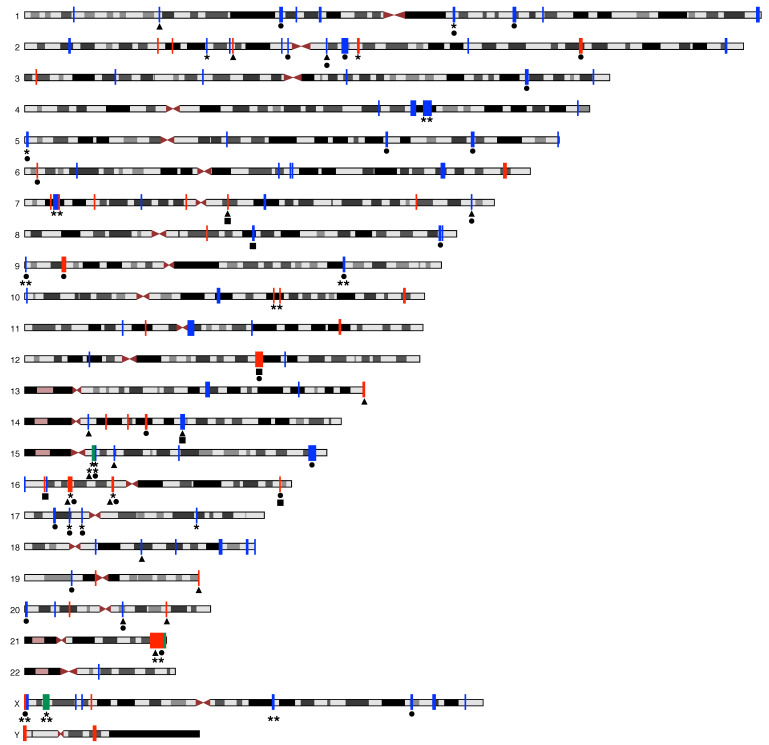
Overview of the copy number variations (CNV) of unknown significance (CNV-US) and the relevant results from our comprehensive re-analysis on a schematic chromosome map. Deletions are red, duplications are blue, regions containing both deletions and duplications are green. Depicted are CNV-US overlapping with a pathogenic region (*), recurrent regions in the study cohort (**), CNV-US containing one or more candidate congenital heart defect (CHD) protein-coding genes (▲), CNV-US containing one or more lncRNA genes expressed in developing human heart (•), and CNV-US potentially disrupting topologically associated domain (TAD)-related gene-enhancer interactions potentially relevant for CHD (■).

A comprehensive overview of the final results of the explorative re-analysis of the CNV-US is given in Figure 1. Figure 3 depicts all CNV-US with their relevant results on a structured chromosome map.

An overview of the protein-coding gene content, lncRNA genes, VISTA enhancer elements, and TAD interference for all studied CNV-US is given in Supplementary Table S8.

## 4. Discussion

In this retrospective study, we reviewed reported CNVs in an in-house cohort of 270 patients with CHD, and re-evaluated the CNV-US considering preliminary evidence of alternative non-coding regulatory elements and the 3D genomic TAD structures.

The number of SCHD was lower in our cohort than in previously reported series, which could explain the lower yield for pathogenic CNVs (6.8%) than previously reported (12.8% to 18.5%) [26]. The 22q11.2 deletion and Williams-Beuren syndrome were the most frequent SCHD entities.

The CNV-US were nearly equally distributed between the SCHD and ICHD subgroup, with copy number gains more than twice as frequent as copy number losses. Over 20% of the CNV-US was larger than 500 kb, which clearly surpasses the number of large CNVs (>500 kb) in the general population (8%) [12]. Twenty-nine of the reported CNV-US (21%) are <100 kb (three having a borderline size of 98.0 kb, 99.4 kb, and 99.9 kb). According to the in-house reporting protocol CNVs < 100 kb are only reported when containing gene-related sequences, though four of these small CNV-US did not contain any protein-coding sequences at all. At least six CNV-US (4.3%) in our cohort occurred de novo. This number might be underestimated, since segregation data were incomplete for over one third of patients. Warburton et al. reported 8% to 12.7% de novo CNVs in a cohort of probands with conotruncal defects or hypoplastic left heart disease, which was considerably higher than the approximately 2% de novo CNVs in a control cohort of trios unaffected with CHD obtained from the Simons Simplex Collection [64,65]. Altogether, this suggests that de novo CNVs may contribute to cardiac phenotypes, but does not preclude the contribution of inherited CNVs, especially in the view of multifactorial disease mechanisms, subtle subclinical phenotypes such as patent foramen ovale, bicuspid aortic valves or spontaneously closed septal defects, or incomplete penetrance.

Our in silico analysis yielded interesting findings for 52 of the 138 CNV-US. We retained three candidate CHD regions from the SRO: chr9:107409509-107729796, chr21:47591379-47671404, and chrX/Y:61091-437220. Evaluation of the protein-coding gene content, based on dosage sensitivity, expression during heart development, and gene function, yielded 19 candidate CHD genes: *ARFGEF2*, *AUTS2*, *CHAMP1*, *CHD8*, *CYFIP1*, *FERMT2*, *ITCH*, *KMT2C*, *MAPK3*, *MAZ*, *MYH11*, *NASP*, *NPAS2*, *PIK3C3*, *PKNOX1*, *TIA1*, *TJP1*, *TRIM28*, *ZBTB21*. At this time, an exhaustive literature search could not provide conclusive evidence for a certain (causal) relationship of these genes with CHD. LncRNA gene analysis confirmed FENDRR as a CHD candidate gene, which has already previously been linked to heart development [66]. For most other lncRNA genes, their functional role remains to be uncovered, precluding suggestions regarding their role in CHD pathogenesis. We further identified six interesting potential interferences with TADs and associated candidate CHD genes. It must be emphasized that only one of the involved VISTA enhancer elements was shown to be expressed in mouse heart at E11.5. Unfortunately, extensive data of expression at other embryonic time points are not available. Moreover, our analysis is restricted to the limited set of experimentally validated VISTA enhancers, and did not evaluate many other enhancers elements that have been reported by initiatives such as ENCODE (https://www.encodeproject.org/) and ROADMAP (http://www.roadmapepigenomics.org/).

Altogether, our explorative in silico analysis raises interest with regards to different potential CNV pathogenic mechanisms interfering with gene expression of protein-coding genes, either through direct copy number alterations of the coding sequence or through interference with lncRNA genes or the 3D genome structure. Our findings ask for experimental validation in future studies, including in vivo and in vitro model systems, expression studies and chromosome conformation capture based analyses such as Hi-C [67,68]. The latter technique has the advantage of allowing both the detection and the interpretation of CNVs [69]. With time, a more complete annotation of both the coding and non-coding genome, and a better understanding of the complex gene regulatory landscape, will aid in estimating the phenotypic consequences of both de novo and (combinations of) inherited variants with smaller individual effects [68].

In patients with SCHD, the diagnostic yield of 19.5% underscores the need for continued molecular karyotyping in a clinical setting. In patients with ICHD, the diagnostic yield remains disputable, mainly due to the large amount of CNV-US identified. In this study, we identified 52 CNV-US of interest, of which 16 CNV-US occurred in 14 SCHD cases (16.1%) and 36 CNV-US occurred in 32 ICHD cases (17.5%). Our in silico analysis shows that the mechanisms affecting non-coding sequences, if confirmed, might substantially contribute to CHD, and will likely increase the diagnostic yield of molecular karyotyping in both SCHD and ICHD. Meanwhile, counseling CNV-US remains challenging. At least, in view of the significantly improved reproductive fitness of patients with CHD, it could be advised to reinterpret the CNV-US at childbearing age. In addition, our data indicate that reporting CNV-US in international databases remains valuable, even if they only contain non-coding sequences.

## 5. Conclusions

We performed a comprehensive reassessment of CNV-US in a large CHD cohort, identifying novel candidate genes for CHD and proving preliminary data for different potentially pathogenic CNV mechanisms interfering with the expression of protein-coding genes, warranting functional validation. Our data call for a reinterpretation of CNV-US in the context of CHD.

## Figures and Tables

**Table 1:** Overlap of CNV-US with known ClinGen Pathogenic or Dosage Sensitive CNV regions.

CNV-US	Known Dosage Sensitive CNV Regions	Interpretation		
Chr1:145388355-145832995dup	1q21.1 recurrent (TAR) region (BP2-BP3, proximal) (ISCA-37428)	99.5% overlap	TS score 1	No definite link with CHD
Chr2:60998688-61093639dup	2p15p16.1 region (ISCA-37408)	2.8% overlap	TS score 1	No link with CHD
Chr2:112650001-112740000del	2q13 recurrent region (ISCA-37496)	5.3% overlap	HI score 2	Linked to CHD; excl CHD candidate gene *BCL2L11*
Chr5:1005001-1290000dup	5p15 terminal (Cri du chat syndrome) region (ISCA-37390)	2.5% overlap	TS score 2	No clear link with CHD
Chr10:84054763-84073574del	10q22.3q23.2 recurrent region (LCR-3/4-flanked) (ISCA-37424)	0.3% overlap	HI score 3	Linked to CHD; excl CHD candidate gene *BMPR1A*
Chr10:88004601-88065186del	10q22.3q23.2 recurrent region (LCR-3/4-flanked) (ISCA-37424)	0.9% overlap	HI score 3	Linked to CHD; excl CHD candidate gene *BMPR1A*
Chr15:22755001-23085000del	15q11.2 recurrent region (BP1-BP2) (ISCA-37448)	97.7% overlap	HI score 2	Link with CHD under debate
Chr15:22765628-23167699del	15q11.2 recurrent region (BP1-BP2) (ISCA-37448)	100% overlap	HI score 2	Link with CHD under debate
Chr15:22765628-23208842dup	15q11.2 recurrent region (BP1-BP2) (ISCA-37448)	100% overlap	TS score 40	-
Chr15:22765628-23208842dup	15q11.2 recurrent region (BP1-BP2) (ISCA-37448)	100% overlap	TS score 40	-
Chr15:24005491-24470088dup	15q11q13 recurrent (PWS/AS) region (BP2-BP3, Class 1) (ISCA-37404)	10.0% overlap	TS score 3	No clear link with CHD
Chr16:14968855-16292181del	16p13.11 recurrent region (BP2-BP3) (ISCA-37415)	100% overlap	HI score 3	Link with CHD under debate
Chr16:29656684-30197290del	16p11.2 recurrent region (proximal, BP4-BP5) (ISCA-37400)	98.3% overlap	HI score 3	Link with CHD under debate
Chr17:15257416-15482813dup	17p12 recurrent (HNPP/CMT1A) region (ISCA-37436)	12.5% overlap	TS score 3	No link with CHD
Chr17:18148172-18662098dup	17p11.2 recurrent (SMS/PLS) region (ISCA-37418)	15.1% overlap	TS score 3	Linked to CHD; excl CHD candidate gene *RAI1*
Chr17:58372095-58588996dup	17q23.1q23.2 recurrent region (ISCA-37501)	10.0% overlap	TS score 2	Link with CHD unclear
ChrX:6467006-8131751del *	Xp22.31 recurrent region (ISCA-37417)	99.3% overlap	HI score 3	No link with CHD
ChrX:6467006-8131751dup *	Xp22.31 recurrent region (ISCA-37417)	99.3% overlap	TS score 40	-
ChrX:7515001-8130000dup	Xp22.31 recurrent region (ISCA-37417)	36.5% overlap	TS score 40	-

HI = haploinsufficiency; TS = triplosensitivity; score 1 = little evidence; score 2 = emerging evidence; score 3 = sufficient evidence; score 40 = dosage sensitivity unlikely; X-chromosomal CNV-US occurring in females are marked with *. All CNV-US were mapped to reference genome GRCh37 (hg19).

**Table 2:** Recurrent CNV-US regions in the study cohort.

CNV-US	Smallest Region of Overlap (SRO) (Protein-Coding Genes)	Interpretation
Chr4:135455435-137460949dup Chr4:135700662-135829279dup	Chr4:135700662-135829279 (no protein-coding genes)	One nearly identical duplication in Decipher, classified ‘likely benign’. Two partially overlapping duplications in the same boundaries in DGV.
Chr7:11221210-12462629dup Chr7:12300173-12462629del	Chr7:12300173-12462629 (*VWDE*)	Two comparable deletions in Decipher, both classified ‘CNV-US’. Multiple comparable CNVs in DGV.
Chr9:195001-405000dup Chr9:210001-540000dup	Chr9:210001-405000 (*DOCK8*)	Multiple comparable CNVs in Decipher, most classified ‘CNV-US’. Multiple comparable CNVs in DGV.
Chr9:107409506-107729796dup Chr9:107409509-107769094dup	**Chr9:107409509-107729769** (*OR13D1, NIPSNAP3A, NIPSNAP3B, ABCA1*)	Four comparable duplications in Decipher, three classified ‘CNV-US’, one classified ‘likely pathogenic’. No comparable CNVs in DGV.
Chr15:22755001-23085000del Chr15:22765628-23167699del Chr15:22765628-23208842dup Chr15:22765628-23208842dup	Chr15:22765628-23085000 (*CYFIP1, NIPA1,NIPA2, TUBGCP5*)	Known ClinGen Pathogenic and Dosage Sensitive CNV region (ISCA-37448).
Chr21:43014314-48090258del Chr21:47591379-47671404dup	**Chr21:47591379-47671404** (*SPATC1L, LSS, MCM3AP*)	No comparable CNVs in Decipher. No comparable CNVs in DGV.
ChrX:61091-437220del * ChrY:61091-819199del	**ChrX/Y:61091-437220** (*PLCXD1, GTPBP6, PPP2R3B*)	No comparable CNVs in Decipher. No comparable CNVs in DGV.
ChrX:6467006-8131751dup * ChrX:6467006-8131751del * ChrX:7515001-8130000dup	ChrX:7515001-8130000 (*VCX, PNPLA4*)	Multiple comparable CNVs in Decipher, all but one classified ‘CNV-US’. Few comparable CNVs in DGV.
ChrX:130610000-130950000dup ChrX:130631863-130960558dup *	ChrX:130631863-130950000 (*OR13H1*)	Multiple comparable CNVs in Decipher, most classified ‘CNV-US’ or ‘benign’. No comparable CNVs in DGV.

Decipher = Decipher database of CNV entries (https://decipher.sanger.ac.uk/, accessed on 22 March 2021); DGV = Database of Genomic Variants (http://dgv.tcag.ca/dgv/app/home, accessed on 22 March 2021); Potential candidate CHD regions are marked in bold. X-chromosomal CNV-US occurring in females are marked with *. All CNV-US were mapped to reference genome GRCh37 (hg19).

## Data Availability

The data presented in this study are available in the manuscript or in the Supplementary Materials, or can be obtained from the authors upon written request to the corresponding author.

## Supplementary Materials

The following are available online at https://www.mdpi.com/article/10.3390/genes12071048/s1, Table S1: CHD-related gene panels, Table S2: Heart defects in the study cohort, Table S3: Pathogenic CNVs in the study cohort, Table S4: CNV-US in the study cohort and subgroups, Table S5: Candidate CHD protein-coding genes in CNV-US, Table S6: Overview of the characteristics of protein-coding genes in CNV-US and TADs of interest, Table S7: LncRNA genes of interest in CNV-US, Table S8: Overview of the studied characteristics per CNV-US.
