# Supplementary material for: A Reassessment of Copy Number Variations in Congenital Heart Defects: Picturing the Whole Genome
Source: Genes (Basel). 2021 Jul 8;12(7):1048. doi: 10.3390/genes12071048 (PMC8304049; doi:10.3390/genes12071048)
Supplement: Supplementary file 1 [file genes-12-01048-s001.zip › genes-1273098-SI/Table S2 S3 S4 S5 S7.pdf]

A Reassessment of Copy Number Variations in Congenital Heart Defects

Picturing the whole genome

Ilse Meerschaut, Sarah Vergult, Annelies Dheedene, Björn Menten, Katya De Groote, Hans De Wilde, Laura Muiño Mosquera, Joseph Panzer, Kristof Vandekerckhove, Paul J. Coucke, Daniël De Wolf, Bert Callewaert.

Correspondence to: Bert Callewaert, Center for Medical Genetics, Ghent University Hospital, C. Heymanslaan 10, 9000 Ghent, Belgium. Email: Bert.Callewaert@UGent.be. Phone: +32 9 332 3603.

Supplemental Table S2. Heart defects in the study cohort

Supplemental Table S3. Pathogenic CNVs in the study cohort

Supplemental Table S4. CNV-US in the study cohort and subgroups

Supplemental Table S5. Candidate CHD protein-coding genes in CNV-US

Supplemental Table S7. LncRNA genes of interest in CNV-US

Supplemental Table S2. Heart defects in the study cohort

	All patients		SCHD patients		ICHD patients	
**All types of congenital heart defects**	**270**	**100%**	**87**	**32.2%**	**183**	**67.8%**
Ventricular septal defect	51	18.9%	27	31.0%	24	13.1%
Aortic coarctation and arch abnormality	42	15.6%	11	12.6%	31	16.9%
Tetralogy of Fallot	39	14.4%	10	11.5%	29	15.8%
Transposition of the great arteries	37	13.7%	0	0%	37	20.2%
Functionally univentricular heart	27	10.0%	10	11.5%	17	9.3%
Atrial septal defect	14	5.2%	8	9.2%	6	3.3%
Left ventricular outflow tract abnormality	11	4.1%	4	4.6%	7	3.8%
Double outlet right ventricle	11	4.1%	2	2.3%	9	4.9%
Right ventricular outflow tract abnormality	8	3.0%	2	2.3%	6	3.3%
Atrioventricular septal defect	7	2.6%	2	2.3%	5	2.7%
Isomerism	6	2.2%	6	6.9%	0	0%
Pulmonary vein abnormality	5	1.9%	1	1.1%	4	2.2%
Ebstein malformation	4	1.5%	3	3.4%	1	0.5%
Truncus arteriosus	4	1.5%	1	1.1%	3	1.6%
Pulmonary atresia intact septum	2	0.7%	0	0%	2	1.1%
Congenital corrected transposition	1	0.4%	0	0%	1	0.5%
Cor triatriatum	1	0.4%	0	0%	1	0.5%

Supplemental Table S3. Pathogenic CNVs in the study cohort

Pathogenic copy number variants in individuals with SCHD	Type of congenital heart defect
22q11.2 recurrent (DGS/VCFS) region (proximal, A-D) (ISCA-37446) – deletion (MIM 188400)	Atrial septal defect
22q11.2 recurrent (DGS/VCFS) region (proximal, A-D) (ISCA-37446) – deletion (MIM 188400)	Ventricular septal defect
22q11.2 recurrent (DGS/VCFS) region (proximal, A-D) (ISCA-37446) – deletion (MIM 188400)	Ventricular septal defect
22q11.2 recurrent (DGS/VCFS) region (proximal, A-D) (ISCA-37446) – deletion (MIM 188400)	Tetralogy of Fallot
22q11.2 recurrent (DGS/VCFS) region (proximal, A-D) (ISCA-37446) – deletion (MIM 188400)	Tetralogy of Fallot
22q11.2 recurrent (DGS/VCFS) region (proximal, A-D) (ISCA-37446) – deletion (MIM 188400)	Tetralogy of Fallot
22q11.2 recurrent (DGS/VCFS) region (proximal, A-D) (ISCA-37446) – deletion (MIM 188400)	Functionally univentricular heart
22q11.2 recurrent region (central, B/C-D) (ISCA-37516) - deletion	Ventricular septal defect
22q11.2 recurrent region (distal type I, D-E/F) (ISCA-37397) – deletion (MIM 611867)	Aortic coarctation
22q11.2 recurrent (DGS/VCFS) region (proximal, A-D) (ISCA-37446) – duplication (MIM 608363)	Ventricular septal defect
7q11.23 recurrent (Williams-Beuren syndrome) region (ISCA-37392) – deletion (MIM 194050)	LVOT abnormality
7q11.23 recurrent (Williams-Beuren syndrome) region (ISCA-37392) – deletion (MIM 194050)	LVOT abnormality
7q11.23 recurrent (Williams-Beuren syndrome) region (ISCA-37392) – deletion (MIM 194050)	LVOT abnormality
7q11.23 recurrent (Williams-Beuren syndrome) region (ISCA-37392) – deletion (MIM 194050)	LVOT abnormality
17q21.3 recurrent region (ISCA-37420) – deletion (MIM 610443)	Atrial septal defect
17p11.2 recurrent (SMS/PLS) region (ISCA-37418) - duplication (MIM 610883)	Functionally univentricular heart
4p16.3 terminal (Wolf Hirschhorn syndrome) region (ISCA-37429) – deletion (MIM 194190)	Atrial septal defect

All CNV-US were mapped to reference genome GRCh37 (hg19)

Supplemental Table S4. CNV-US in the study cohort and subgroups

CNV-US	Subgroup	CNV-US	Subgroup
chr1:17241750-17393588dup	ICHD	chr10:88004601-88065186del	SCHD
**chr1:45993451-46050273dup**	SCHD	chr10:128458646-128823028del	ICHD
**chr1:86488537-87236743dup**	SCHD	chr11:33192074-33421736dup	SCHD
chr1:92464376-92606671dup	SCHD	chr11:40618648-40742235del	ICHD
chr1:100110001-100545000dup	ICHD	chr11:55050707-56931087dup	ICHD
**chr1:145388355-145832995dup**	SCHD	chr11:76670039-76777036dup	ICHD
**chr1:165562486-166482444dup**	ICHD	chr11:106050001-106260000del	ICHD
chr1:175448847-175726807dup	ICHD	chr12:21547800-21644501dup	ICHD
chr1:247740001-248565000dup	ICHD	**chr12:77616529-79689969del**	ICHD
chr2:14701626-15159618dup	SCHD	chr12:87900001-88080000dup	SCHD
chr2:44507915-44579904del	ICHD	chr13:61711359-62962420dup	SCHD
chr2:49545371-49629804del	ICHD	chr13:92966240-93046389dup	ICHD
chr2:60998688-61093639dup	ICHD	**chr13:114843912-115105238del**	ICHD
chr2:68685528-68915775dup	ICHD	**chr14:21902947-21909605dup**	ICHD
**chr2:70439112-70488413del**	ICHD	chr14:27650380-27799841del	SCHD
chr2:86447244-86537744dup	ICHD	chr14:35110121-35167580del	ICHD
**chr2:88257759-89016165trip**	ICHD	**chr14:41234593-41536386del**	ICHD
**chr2:101521192-101659259dup**	SCHD	**chr14:53326238-54313294dup**	ICHD
**chr2:106878050-108441524dup**	SCHD	**chr15:22755001-23085000del**	ICHD
chr2:112650001-112740000del	ICHD	**chr15:22765628-23208842dup**	ICHD
chr2:149079154-149313760dup	SCHD	**chr15:22765628-23167699del**	ICHD
**chr2:186857026-187327501del**	ICHD	**chr15:22765628-23208842dup**	ICHD
chr2:236075658-236458404trip	ICHD	chr15:24005491-24470088dup	ICHD
chr3:4337407-4357235del	SCHD	**chr15:29872834-30019045dup**	ICHD
chr3:30882231-30974326dup	ICHD	chr15:51739647-51791693trip	ICHD
chr3:60431642-60690267dup	SCHD	**chr15:95701920-97765966dup**	SCHD
chr3:108850038-108948062dup	SCHD	chr16:258392-462341dup	SCHD
**chr3:169521817-170020490dup**	ICHD	**chr16:5030678-5546770del**	ICHD
chr3:192385902-192488655dup	SCHD	chr16:7830001-8055000dup	ICHD
chr4:120033531-120113986dup	ICHD	**chr16:14968855-16292181del**	ICHD
chr4:131146744-132525235dup	ICHD	**chr16:29656684-30197290del**	ICHD
chr4:135455435-137460949dup	ICHD	**chr16:86409444-86509316del**	ICHD
chr4:135700662-135829279dup	SCHD	**chr17:9981738-10410275dup**	SCHD
chr4:187171324-187294087dup	ICHD	**chr17:15257416-15482813dup**	SCHD
chr4:187333416-187518707dup	ICHD	**chr17:18148172-18662098dup**	SCHD
**chr5:1005001-1290000dup**	SCHD	chr17:58372095-58588996dup	ICHD
chr5:68595931-68635696trip	SCHD	chr18:23904986-23996644dup	SCHD
**chr5:122233184-122489348dup**	ICHD	**chr18:39451438-39554147dup**	ICHD
**chr5:151095022-151482286dup**	ICHD	chr18:50883661-51084413dup	SCHD
chr5:180119112-180218463dup	ICHD	chr18:65727205-66532484dup	SCHD
**chr6:4269700-4465244del**	ICHD	chr18:75104351-75387552dup	ICHD
chr6:17670001-17805000dup	SCHD	chr18:77733413-77762403dup	ICHD

chr6:86004058-86204900dup	SCHD	**chr19:15850613-15978604dup**	SCHD
chr6:90315001-90510000dup	ICHD	chr19:23778647-23950181del	ICHD
chr6:91188432-91351703dup	SCHD	**chr19:58980970-59092515del** mos	SCHD
chr6:140957317-141785879dup	ICHD	**chr20:67778-439387dup**	ICHD
chr6:162130733-162799322del	SCHD	chr20:9736328-9968799dup	ICHD
chr7:9600001-9780000del	SCHD	chr20:14928568-15182995del	SCHD
chr7:11221210-12462629dup	SCHD	**chr20:32820001-33045000trip**	ICHD
chr7:12300173-12462629del	ICHD	**chr20:47471691-47625126del**	SCHD
chr7:24335739-24416109del	ICHD	**chr21:43014314-48090258del**	ICHD
chr7:40152590-40256612dup	ICHD	**chr21:47591379-47671404dup**	ICHD
chr7:55305001-55440000del	ICHD	chr22:25102007-25247453dup	ICHD
**chr7:69330737-69584704del**	SCHD	**chrX:61091-437220del**	ICHD *
chr7:81972080-82369067dup	ICHD	chrX:481940-638810dup	ICHD
chr7:133070210-133120414del	ICHD	chrX:908162-1259089trip	ICHD
**chr7:151800001-151905000dup**	ICHD	chrX:6467006-8131751dup	ICHD *
chr8:62035047-62172054del	ICHD	chrX:6467006-8131751del	SCHD *
**chr8:77880001-78240000dup**	ICHD	chrX:7515001-8130000dup	ICHD
chr8:140830000-141140000dup	SCHD	chrX:17388654-17417102dup	SCHD *
**chr8:142131562-142255482dup**	SCHD	chrX:19491688-19601181dup	ICHD
chr9:195001-405000dup	ICHD	chrX:22944987-23031936del	SCHD
**chr9:210001-540000dup**	ICHD	chrX:83974189-84378746dup	ICHD *
**chr9:12515544-13415849del**	SCHD	**chrX:130610000-130950000dup**	ICHD
**chr9:107409506-107729796dup**	ICHD	**chrX:130631863-130960558dup**	ICHD *
**chr9:107409509-107769094dup**	ICHD	chrX:138183513-138735042dup	ICHD *
chr10:1201103-1273934dup	ICHD	chrX:148886475-149084873dup	ICHD *
chr10:65690000-66491000dup	SCHD	**chrY:61091-819199del**	ICHD
chr10:84054763-84073574del	ICHD	chrY:23730419-24426917del	SCHD

CNV-US potentially related to CHD pathogenesis based on the elements studied are marked in bold.
X-chromosomal CNV-US occurring in females are marked with *.
All CNV-US were mapped to reference genome GRCh37(hg19).

Supplemental Table S5. Candidate CHD protein-coding genes in CNV-US

CNV-US	Protein-coding gene	Interpretation	
Chr1:45993451-46050273dup	*NASP*	Chromatin	-
Chr2:70439112-70488413del	*TIA1*	FGF	-
Chr2:101521192-101659259dup	*NPAS2*	TF	-
Chr7:69330737-69584704del	*AUTS2*	Ras, histone	Potentially linked to mild heart defects[1]
Chr7:151800001-151905000dup	*KMT2C*	Histone	Kleefstra syndrome 2 (MIM 617768) ; candidate CHD gene[2]
Chr13:114843912-115105238del	*CHAMP1*	TF	-
Chr14:21902947-21909605dup	*CHD8*	CHD panel, WNT, chromatin	Interacts with CHD7[3] ; expressed during rat postnatal cardiac development[4]
Chr14:53326238-54313294dup	*FERMT2*	WNT, TGFβ, sarcomere	-
Chr15:22755001-23085000del	*CYFIP1*	Ras	-
Chr15:22765628-23167699del	*CYFIP1*	Ras	-
Chr15:29872834-30019045dup	*TJP1*	Hippo	Role in formation of gap junctions in rat cardiomyocytes[5] ; association of ZO-1 with Cx43 in cardiac myocytes[6] ; Cx43 knockout mice exhibit conotruncal malformations and coronary artery defects[7]
Chr16:14968855-16292181del	*MYH11*	CHD panel	Related to thoracic aortic aneurysm / aortic dissection and PDA[8] ; role in mesenchymal and endothelial cell differentiation, associated with valvulogenesis and endothelial to mesenchymal transition[9]
Chr16:29656684-30197290del	*MAPK3*	BMP, FGF, histone	Linked to cardiac hypertrophy in transgenic mouse[10]
	MAZ	TF	Transcriptional regulator of muscle-specific genes in cardiac myocytes[11]
Chr18:39451438-39554147dup	*PIK3C3*	Cilium	Essential role in regulating autophagy and heart function[12]
Chr19:58980970-59092515del	*TRIM28*	Ras	Regulator of cardiomyocyte differentiation in murine embryonic stem cells[13] ; regulates sprouting angiogenesis in zebrafish[14]
Chr20:32820001-33045000trip	*ITCH*	Notch	-
Chr20:47471691-47625126del	*ARFGEF2*	Ras, cilium	Linked to vascular development in zebrafish[15]
Chr21:43014314-48090258del	*PKNOX1*	TF	PBX-related genes are candidates for CHD[16]
	ZBTB21	TF	Potential role in CHD pathogenesis in Down syndrome[17]

TF = transcription factor ; BMP = bmp signaling pathway ; WNT = wnt signaling pathway ; Notch = notch signaling pathway ; TGFβ = transforming growth factor β receptor signaling pathway ; Hippo = hippo signaling ; FGF = fibroblast growth factor receptor signaling pathway ; Ras = ras protein signal transduction ; Histone = histone modification ; Chromatin = chromatin remodeling.
All CNV-US were mapped to reference genome GRCh37 (hg19).

1. Beunders G, van de Kamp J, Vasudevan P, et al. A detailed clinical analysis of 13 patients with AUTS2 syndrome further delineates the phenotypic spectrum and underscores the behavioural phenotype. J Med Genet. 2016;53(8):523-532.
2. Szot JO, Cuny H, Blue GM, et al. A Screening Approach to Identify Clinically Actionable Variants Causing Congenital Heart Disease in Exome Data. Circ Genom Precis Med. 2018;11(3):e001978.
3. Batsukh T, Pieper L, Koszucka AM, et al. CHD8 interacts with CHD7, a protein which is mutated in CHARGE syndrome. Hum Mol Genet. 2010;19(14):2858-2866.
4. Shanks MO, Lund LM, Manni S, Russell M, Mauban JR, Bond M. Chromodomain helicase binding protein 8 (Chd8) is a novel A-kinase anchoring protein expressed during rat cardiac development. PLoS One. 2012;7(10):e46316.
5. Wu JC, Tsai RY, Chung TH. Role of catenins in the development of gap junctions in rat cardiomyocytes. J Cell Biochem. 2003;88(4):823-835.
6. Toyofuku T, Yabuki M, Otsu K, Kuzuya T, Hori M, Tada M. Direct association of the gap junction protein connexin-43 with ZO-1 in cardiac myocytes. J Biol Chem. 1998;273(21):12725-12731.
7. Rhee DY, Zhao XQ, Francis RJ, Huang GY, Mably JD, Lo CW. Connexin 43 regulates epicardial cell polarity and migration in coronary vascular development. Development. 2009;136(18):3185-3193.

8. Zhu L, Vranckx R, Khau Van Kien P, et al. Mutations in myosin heavy chain 11 cause a syndrome associating thoracic aortic aneurysm/aortic dissection and patent ductus arteriosus. Nat Genet. 2006;38(3):343-349.
9. Lu CC, Liu MM, Clinton M, Culshaw G, Argyle DJ, Corcoran BM. Developmental pathways and endothelial to mesenchymal transition in canine myxomatous mitral valve disease. Vet J. 2015;206(3):377-384.
10. Lorenz K, Schmitt JP, Schmitteckert EM, Lohse MJ. A new type of ERK1/2 autophosphorylation causes cardiac hypertrophy. Nat Med. 2009;15(1):75-83.
11. Himeda CL, Ranish JA, Hauschka SD. Quantitative proteomic identification of MAZ as a transcriptional regulator of muscle-specific genes in skeletal and cardiac myocytes. Mol Cell Biol. 2008;28(20):6521-6535.
12. Jaber N, Dou Z, Chen JS, et al. Class III PI3K Vps34 plays an essential role in autophagy and in heart and liver function. Proc Natl Acad Sci U S A. 2012;109(6):2003-2008.
13. Gan L, Schwengberg S, Denecke B. Transcriptome analysis in cardiomyocyte-specific differentiation of murine embryonic stem cells reveals transcriptional regulation network. Gene Expr Patterns. 2014;16(1):8-22.
14. Wang Y, Singh AR, Zhao Y, et al. TRIM28 regulates sprouting angiogenesis through VEGFR-DLL4-Notch signaling circuit. Faseb j. 2020;34(11):14710-14724.
15. Lu FI, Wang YT, Wang YS, Wu CY, Li CC. Involvement of BIG1 and BIG2 in regulating VEGF expression and angiogenesis. Faseb j. 2019;33(9):9959-9973.
16. Arrington CB, Dowse BR, Bleyl SB, Bowles NE. Non-synonymous variants in pre-B cell leukemia homeobox (PBX) genes are associated with congenital heart defects. Eur J Med Genet. 2012;55(4):235-237.
17. Sailani MR, Makrythanasis P, Valsesia A, et al. The complex SNP and CNV genetic architecture of the increased risk of congenital heart defects in Down syndrome. Genome Res. 2013;23(9):1410-1421.

Supplemental Table S7. LncRNA genes of interest in CNV-US

CNV-US	lncRNA of interest with TPM 2 (TPM10) in developing human heart
Chr1:86488537-87236743dup	lnc-ODF2L-32, lnc-SELENOF-2
Chr1:145388355-145832995dup	LIX1L-AS1
Chr1:165562486-166482444dup	lnc-TMCO1-1, lnc-UCK2-1
Chr2:88257759-89016165trip	lnc-RPIA-2
Chr2:101521192-101659259dup	lnc-TBC1D8-7
Chr2:106878050-108441524dup	ST6GAL2-IT1
Chr2:186857026-187327501del	LINC01473
Chr3:169521817-170020490dup	SEC62-AS1
Chr5:1005001—1290000dup	**lnc-SLC12A7-5**
Chr5:122233184-122489348dup	lnc-PPIC-1
Chr5:151095022-151482286dup	lnc-ATOX1-1, lnc-G3BP1-1, lnc-GLRA1-1
Chr6:4269700-4465244del	**lnc-ECI2-2**
Chr7:151800001-151905000dup	lnc-KMT2C-1
Chr8:142131562-142255482dup	lnc-SLC45A4-2, lnc-SLC45A4-3
Chr9:210001-540000dup	lnc-DOCK8-1
Chr9:12515544-13415849del	lnc-NFIB-1
Chr9:107409506-107729796dup	lnc-NIPSNAP3B-1
Chr9:107409509-107769094dup	lnc-NIPSNAP3B-1
Chr12:77616529-79689969del	lnc-ZDHHC17-20
Chr14:41234593-41536386del	LINC02315
Chr15:22765628-23208842dup	**lnc-NIPA1-2**
Chr15:22765628-23167699del	**lnc-NIPA1-2**
Chr15:22765628-23208842dup	**lnc-NIPA1-2**
Chr15:95701920-97765966dup	LINC01197, LINC02157, lnc-NR2F2-1, lnc-NR2F2-2, lnc-NR2F2-15, lnc-PGPEP1L-60, NR2F2-AS1
Chr16:14968855-16292181del	lnc-ABCC1-1, lnc-C16orf45-1, **lnc-C16orf45-4**
Chr16:29656684-30197290del	lnc-ASPHD1-1, lnc-CDIPT-1, lnc-PPP4C-10, lnc-PPP4C-11
Chr16:86409444-86509316del	FENDRR
Chr17:9981738-10410275dup	lnc-ADPRM-2
Chr17:15257416-15482813dup	lnc-CDRT4-4
Chr17:18148172-18662098dup	lnc-MIEF2-1, **lnc-TBC1D28-1**, lnc-TBC1D28-6
Chr19:15850613-15978604dup	UCA1
Chr20:67778-439387dup	**NRSN2-AS1**
Chr20:32820001-33045000trip	ITCH-IT1, lnc-EIF2S2-4
Chr21:43014314-48090258del	COL18A1-AS1, COL18A1-AS2, DIP2A-IT1, ITGB2-AS1, **LINC00205**, LINC00316, LINC00479, LINC01424, LINC01679, lnc-C2CD2-1, lnc-COL6A1-1, lnc-CRYAA-2, **lnc-FAM207A-2**, lnc-FAM207A-10, **lnc-LRRC3-5**, **lnc-LSS-1**, lnc-PTTG1IP-7, lnc-RSPH1-1, **lnc-SLC19A1-7**, lnc-WDR4-1, lnc-WDR4-2, lnc-WDR4-4, lnc-YBEY-5, LRRC3-DT, PICSAR, TSPEAR-AS1, TSPEAR-AS2
Chr21:47591379-47671404dup	lnc-YBEY-5
ChrX:61091-437220del	**LINC00685**, lnc-PLCXD1-5, lnc-PLCXD1-6
ChrX:130610000-130950000dup	FIRRE
ChrX:130631863-130960558dup *	FIRRE

lncRNA genes expressed in developing human heart tissue reaching TPM10 are marked in bold.
X-chromosomal CNV-US occurring in females are marked with *.
CNV-US were mapped to reference genome GRCh37 (hg19).
